# Dog-Mediated Human Rabies Death, Haiti, 2016

**DOI:** 10.3201/eid2211.160826

**Published:** 2016-11

**Authors:** Ryan M. Wallace, Melissa D. Etheart, Jeff Doty, Ben Monroe, Kelly Crowdis, Pierre Dilius Augustin, Jesse Blanton, Natael Fenelon

**Affiliations:** Centers for Disease Control and Prevention, Atlanta, Georgia, USA (R.M. Wallace, J. Doty, B. Monroe, J. Blanton);; Centers for Disease Control and Prevention, Port-au-Prince, Haiti (M.D. Etheart);; Christian Veterinary Mission, Port-au-Prince (K. Crowdis);; Ministry of Agriculture, Natural Resources and Rural Development, Port-au-Prince (P. Dilius Augustin);; Pan American Health Organization, Port-au-Prince (N. Fenelon)

**Keywords:** rabies, Haiti, zoonoses, viruses, rabies vaccinations, hydrophobia, veterinary medicine, animal diseases, dogs

## Abstract

Haiti has experienced numerous barriers to rabies control over the past decades and is one of the remaining Western Hemisphere countries to report dog-mediated human rabies deaths. We describe the circumstances surrounding a reported human rabies death in 2016 as well as barriers to treatment and surveillance reporting.

Rabies kills 59,000 persons each year worldwide, more than any other zoonotic disease (*1*). In the Western Hemisphere, deaths caused by dog-mediated human rabies have been nearly eliminated. However, these deaths still occur in Haiti, where researchers estimate that up to 130 persons die from dog-mediated rabies each year (*1*). Disease surveillance in Haiti since the 2010 earthquake has improved, but the capacity for detecting and responding to rabies cases is still limited. We describe an investigation of a suspected human rabies case, including the patient’s clinical signs and symptoms and the healthcare and public health response to dog-mediated human rabies. We also highlight challenges faced by Haiti’s public health system.

## Case Report

On January 14, 2016, a woman with behavioral changes and hydrophobia visited a regional hospital in Cap-Haïtien, Nord Department, Haiti. Her husband reported that a dog had bitten her 3 months before symptoms developed. A local clinic treated the wound on the day the bite occurred but did not offer rabies vaccination. Healthcare workers at the regional hospital made a presumptive diagnosis of rabies but were unable to offer palliative care. The couple left without providing additional contact information. The hospital administrator reported the suspected rabies case to the national Department of Epidemiology and Laboratory Research (DELR), as required by Haiti’s national surveillance system. Without contact information, DELR was unable to investigate further.

Haiti’s Ministry of Health (*Ministère de la Santé Publique et de la Population* [MSPP]), with assistance from the US Centers for Disease Control and Prevention (CDC), has developed a robust surveillance system for 44 conditions, 13 of which are immediately reportable, including suspected human rabies. Under this surveillance system, health alerts for suspected human rabies cases are investigated to confirm clinical cases of rabies, identify persons or animals exposed to a rabid animal, and identify healthcare and community contacts of the person suspected of having rabies. Since February 2015, CDC has assisted DELR in 3 human rabies investigations, which identified 27 rabies-exposed persons in addition to patients. 

The person with suspected rabies in this case report was not admitted to the hospital, and no contact information was obtained. Therefore, public health investigators could not determine her health outcome, gather potential human and animal exposures, or complete classification of this case on the basis of the World Health Organization’s clinical case definition for rabies (*2*).

On March 14, 2016, three months after the woman with suspected rabies had visited the regional hospital, a CDC-trained veterinarian who was conducting a rabies survey among mongooses was alerted by community members to a potential human rabies death. Initial reports led the veterinarian to believe that the person who died was the same woman who sought care at the Cap-Haïtien hospital. A team of healthcare workers from CDC and the Pan American Health Organization (PAHO) had already planned training on integrated bite case management (IBCM) in this area. In addition to the training, during March 30–April 10, CDC and PAHO assisted Haiti’s Ministry of Agriculture, Natural Resources and Rural Development (MARNDR) and DELR in investigating the suspected human rabies case.

On April 5, 2016, the investigation team conducted a verbal autopsy with the decedent’s husband. The investigation confirmed that the person who died was a 54-year-old woman who was bitten on the left hand on November 30, 2015, while fending off a dog that was acting aggressively toward her goats. The woman visited a local healer, who administered 1 shot of an unknown substance. Except for residual pain in the hand, the woman remained healthy until January 10, 2016, when her husband recognized signs of confusion; notably, she had placed common household items in unusual locations. During January 11–13, fevers, hypersalivation, agitation, and incoherent speech developed. On January 14, the woman accused her husband of trying to kill her when he offered her water (presumed hydrophobia). On that day, the husband and wife traveled to a health clinic and were immediately referred to the regional referral hospital in Cap-Haïtien. The husband reported that palliative care was denied, and they left the hospital without providing contact information. The wife died later that night. According to WHO clinical rabies case definitions, the woman’s illness was a probable rabies case.

The investigators verified that neighbors had killed an abnormally aggressive dog on approximately November 30, 2015. Neighbors reported that the dog had attempted to bite several persons, but it was killed without further human exposures. The dog had bitten 1 pig, which could not be located because of the delay between the bite event and the case investigation.

## Conclusions

Few cases of rabies in Haiti are reported to health authorities; in 2015, only 7 cases were documented, 5 of which were detected through the veterinary sector. Lack of recognition of rabies has been attributed to low awareness, unique cultural beliefs, and a high incidence of numerous conditions (i.e., cerebral malaria, meningitis, viral encephalitis, and tetanus) that may confound rabies diagnosis (*3**,**4*). Furthermore, diagnosis of human rabies is not performed in Haiti due to limitations in diagnostic capacity and cultural aversion to collection of postmortem samples. 

The healthcare team identified no other human rabies exposures in this investigation. Given the delay in investigating this case, if any persons had been exposed, they likely would have already succumbed to rabies, underscoring the importance of timely reporting and investigation. In 2015, in an effort to improve healthcare provider recognition of rabies cases and surveillance reporting, PAHO and MSPP developed a rabies training course for healthcare providers. Trainings in Cap-Haïtien are planned for 2016.

Although animal rabies is a reportable condition to MARNDR and bite events are reportable to MSPP, neither the rabid dog nor the bite event were reported in this situation. A 2014 survey estimated that 95,000 animal bites occur annually in Haiti (1% bite rate) (*5*). However, only 6,500 bites (6.8% of estimated bites) were reported through the national surveillance program that year. To improve bite detection and healthcare-seeking behaviors, CDC and PAHO collaborated with MARNDR, DELR, and MSPP to develop an IBCM system to assist in reporting bites to MARNDR for animal investigation. Results are reported to bite victims and to the responsible healthcare sector. Since its inception, the IBCM system has increased detection of animal rabies cases 18-fold and improved patient healthcare-seeking behavior. However, the system’s success depends on reliable and timely reporting. The IBCM program is now operational in 3 of Haiti’s 10 departments but is not yet available in Cap-Haïtien. Therefore, even if the bite had been reported through appropriate surveillance channels, follow-up likely would not have occurred. To improve reporting in Nord Department (Cap-Haïtien), the investigation team has trained 11 veterinary professionals to use the IBCM program (Figure). 

**Figure:**
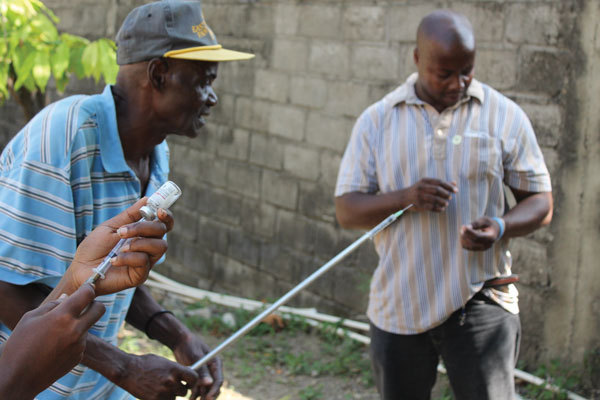
A team consisting of workers from the US Centers of Disease Control and Prevention; the Pan American Health Organization; Haiti’s Ministry of Agriculture, Natural Resources and Rural Development; and Christian Veterinary Mission trained 11 veterinary professionals on principles of animal rabies surveillance. Here, trainees gain experience drawing up sedative medications into a pole syringe, which is used to sedate suspected rabid animals from a safe distance. (Photograph courtesy of R.M. Wallace.)

Haiti has made considerable strides in controlling dog-mediated human rabies deaths through efforts such as dog vaccination, the implementation of the IBCM system, and medical provider training. These advances have been made through collaborative work with Haiti’s government institutions and international partners. Through the continued support and expansion of these programs, cases like the one reported in this article will eventually be eliminated. This case is an unfortunate reminder that dog-mediated human rabies deaths continue to occur in some Western Hemisphere countries; however, this death provided stimulus for training local health officials and served as a reminder of why Haitians and international partners seek elimination of this disease.
